# Risk Factors for Surgical Site Infection After Lower Limb Revascularization Surgery in Adults With Peripheral Artery Disease: Protocol for a Systematic Review and Meta-analysis

**DOI:** 10.2196/28759

**Published:** 2021-09-16

**Authors:** Derek J Roberts, Sudhir K Nagpal, Henry T Stelfox, Tim Brandys, Vicente Corrales-Medina, Luc Dubois, Daniel I McIsaac

**Affiliations:** 1 Department of Surgery Faculty of Medicine University of Ottawa Ottawa, ON Canada; 2 The O'Brien Institute for Public Health, University of Calgary Calgary, AB Canada; 3 Department of Critical Care Medicine Faculty of Medicine University of Calgary Calgary, AB Canada; 4 Department of Medicine University of Ottawa Ottawa, ON Canada; 5 Department of Surgery Western University London, ON Canada; 6 Department of Anesthesia and Pain Medicine University of Ottawa Ottawa, ON Canada

**Keywords:** lower limb revascularization surgery, peripheral artery disease, risk factors, surgical site infection, systematic review

## Abstract

**Background:**

Surgical site infections (SSIs) are common, costly, and associated with increased morbidity and potential mortality after lower limb revascularization surgery (ie, arterial bypass, endarterectomy, and patch angioplasty). Identifying evidence-informed risk factors for SSI in patients undergoing these surgeries is therefore important.

**Objective:**

The aim of this study is to conduct a systematic review and meta-analysis of prognostic studies to identify, synthesize, and determine the certainty in the cumulative evidence associated with reported risk factors for early and delayed SSI after lower limb revascularization surgery in adults with peripheral artery disease.

**Methods:**

We will search MEDLINE, Embase, the seven databases in Evidence-Based Medicine Reviews, review articles identified during the search, and included article bibliographies. We will include studies of adults (aged ≥18 years) with peripheral artery disease that report odds ratios, risk ratios, or hazard ratios adjusted for the presence of other risk factors or confounding variables and relating the potential risk factor of interest to the development of SSI after lower limb revascularization surgery. We will exclude studies that did not adjust for confounding, exclusively examined certain high-risk patient cohorts, or included >20% of patients who underwent surgery for indications other than peripheral artery disease. The primary outcomes will be early (in-hospital or ≤30 days) SSI and Szilagyi grade I (cellulitis involving the wound), grade II (infection involving subcutaneous tissue), and grade III (infection involving the vascular graft) SSI. Two investigators will independently extract data and evaluate the study risk of bias using the Quality in Prognosis Studies tool. Adjusted risk factor estimates with similar definitions will be pooled using DerSimonian and Laird random-effects models. Heterogeneity will be explored using stratified meta-analyses and meta-regression. Finally, we will use the Grading of Recommendations, Assessment, Development, and Evaluation (GRADE) approach to determine certainty in the estimates of association between reported risk factors and the development of SSI.

**Results:**

The protocol was registered in PROSPERO (International Prospective Register of Systematic Reviews). We will execute the peer-reviewed search strategy on June 30, 2021, and then complete the review of titles and abstracts and full-text articles by July 30, 2021, and September 15, 2021, respectively. We will complete the full-text study data extraction and risk of bias assessment by November 15, 2021. We anticipate that we will be able to submit the manuscript for peer review by January 30, 2022.

**Conclusions:**

This study will identify, synthesize, and determine the certainty in the cumulative evidence associated with risk factors for early and delayed SSI after lower limb revascularization surgery in patients with peripheral artery disease. The results will be used to inform practice, clinical practice statements and guidelines, and subsequent research.

**Trial Registration:**

PROSPERO International Prospective Register of Systematic Reviews CRD42021242557; https://www.crd.york.ac.uk/prospero/display_record.php?RecordID=242557

**International Registered Report Identifier (IRRID):**

PRR1-10.2196/28759

## Introduction

### Background

Lower limb revascularization surgeries (ie, arterial bypass, endarterectomy, and patch angioplasty) are costly, high-risk procedures commonly performed in North America and internationally [1-5]. Indications for these procedures include chronic limb-threatening ischemia (ie, peripheral artery disease manifested by ischemic rest pain confirmed by vascular hemodynamic studies, lower limb ulceration, or gangrene) or, less commonly, disabling intermittent vasculogenic claudication that has failed medical management [6]. Although endovascular therapies are increasingly being used to treat patients with these indications, they are not as durable or suited for all patients’ anatomical pattern of peripheral artery disease [7-10]. Therefore, in the United States, approximately 15,000-20,000 lower limb revascularization surgeries are performed each year, and the estimated cost per procedure can exceed US $120,000 [1,2].

Surgical site infections (SSIs) are common and costly; they are also associated with a significantly increased risk of morbidity, limb loss, and potential mortality after lower limb revascularization surgery [11-14]. They represent the leading cause of unplanned and potentially preventable hospital readmissions after vascular surgery [15,16]. After vascular surgery, postoperative complications are commonly classified using the Szilagyi grading system, which includes Szilagyi grade I (cellulitis involving the wound), grade II (infection involving subcutaneous tissue), and grade III (infection involving the vascular graft) SSIs [17-19]. SSI has been estimated to occur in approximately 7% to 8% of patients after infrainguinal bypass surgery (and to result in graft infection in approximately 2%), nearly double the incidence of prolonged (>10 days) hospitalization, and increase the risk-adjusted costs per peripheral vascular surgery by approximately US $7300 (and likely several-fold higher after a deep SSI) [11,12,20]. SSIs involving prosthetic (and sometimes autologous) grafts also often necessitate urgent reoperation for graft excision to prevent pseudoaneurysm formation and catastrophic hemorrhage [21].

Identifying valid, evidence-informed risk factors for SSI after lower limb revascularization surgery is important to assist in deciding which patients may benefit most from interventions designed to prevent them. It may also help in determining the benefit-to-risk profile of performing open over endovascular revascularization in patients who are candidates for both. However, although many studies examining potential risk factors for SSI after lower limb revascularization surgery have been published, some are difficult to access because their titles may not indicate that they examined risk factors for SSI. They are also collectively difficult to interpret, as some have included potentially overlapping data and many are limited by between-study clinical heterogeneity. This heterogeneity includes the recruitment of patients with different indications for surgery (eg, intermittent vasculogenic claudication, chronic limb-threatening ischemia, or even aneurysms and vascular trauma [14]) and examination of different types of lower limb revascularization surgery (eg, ranging from groin-only procedures [22,23] to infrainguinal or aortofemoral bypass [24,25]) or vascular surgery in general [26]. Studies have also either not adjusted for or variably adjusted for potential confounding factors when examining associations between potential risk factors and SSI. Further, some studies have reported on risk factors for specific types of infection (eg, prosthetic graft infection [27] or that requiring reoperation [28]) or for infection or wound complications in general [29]. Finally, others measured their SSI outcomes at different time points ranging from in-hospital to 30 days or longer or defined the severity of SSIs using different classification systems.

### Objectives

We aim to conduct a systematic review and meta-analysis of prognostic studies to identify, synthesize, and determine the certainty in the cumulative evidence associated with reported risk factors for early and delayed SSI after lower limb revascularization surgery in adults with peripheral artery disease. We will also determine whether the strength of association for individual risk factors varies according to different clinical or methodological study characteristics.

## Methods

### Protocol and Role of the Sponsor

We prespecified our methods according to the PRISMA (Preferred Reporting Items in Systematic Reviews and Meta-Analyses) statement [30] and the Meta-Analysis of Observational Studies in Epidemiology proposal [31]. It is reported according to the PRISMA-P (Preferred Reporting Items in Systematic Reviews and Meta-Analysis Protocols) statement (and the completed PRISMA-P checklist is given in Multimedia Appendix 1) [32,33]. The study will follow guidelines for conducting systematic reviews and meta-analyses of prognostic factor studies [34,35]. The protocol is registered on PROSPERO (International Prospective Register of Systematic Reviews; CRD42021242557). The study sponsor, the University of Ottawa, played no role in the development of the protocol.

### Focused Clinical Question

We formulated the study-focused clinical question using the PICOTS (Population, Index Prognostic Factor, Comparison of Prognostic Factors, Outcome, Timing, and Setting) framework for posing clinical questions for systematic reviews of prognostic factor studies [34,36]. The focused clinical question was as follows:

- P: in adults (aged ≥18 years) with peripheral artery disease who underwent lower limb revascularization surgery
- I: which factors increase the risk of SSI or Szilagyi grade I (cellulitis involving the wound), grade II (infection involving subcutaneous tissue), or grade III (infection involving the vascular graft) SSI
- C: over and above other comparator risk and confounding factors for predicting SSI
- OTS: in-hospital or within ≤30 days or longer than 30 days after lower limb revascularization surgery?

### Information Sources

We will search MEDLINE; MEDLINE Epub Ahead of Print, In-Process, and Other Nonindexed Citations; Embase; and the databases contained within Evidence-Based Medicine Reviews (American College of Physicians Journal Club; the Cochrane Central Register of Controlled Trials, the Database of Systematic Reviews, and the Methodology Register Database; the Database of Abstracts of Reviews of Effects; the Health Technology Assessment Database; and the National Health Service Economic Evaluation Database) from their first available dates without restrictions. To identify additional citations, we will also use the PubMed *related articles* feature and manually search bibliographies of included studies and relevant review articles identified during the search.

### Search Strategy

A vascular and endovascular surgeon and epidemiologist with a PhD training in information science and evidence synthesis methods created the initial MEDLINE search strategy, which was refined after input from a medical librarian and information specialist and by adding additional thesaurus or indexing terms when new and relevant citations were located during iterative search strategies. Using a combination of Medical Subject Heading/Emtree terms and keywords, we constructed search filters covering the themes *lower limb revascularization surgery* and *surgical site infection*. After the refined search strategy was created, we submitted it to another medical librarian or information scientist to peer review this search strategy using the Peer Review of Electronic Search Strategies guidelines [37] (our pre–peer-reviewed electronic search strategies are given in Table 1).

**Table 1:** Pre–Peer Review of Electronic Search Strategies database search strategies.

Search theme	Search terms			
	Ovid MEDLINE, PubMed, and Evidence-Based Medicine Reviews		Ovid Embase	
	Exploded MeSH^a^ terms	Title and subject keywords	Exploded Emtree terms	Title and subject keywords
Lower extremity revascularization surgery	Arterial occlusive disease/surgery OR endarterectomy OR ischemia/surgery OR lower extremity/surgery OR peripheral arterial disease/surgery OR peripheral vascular diseases/surgery OR vascular surgical procedures	((iliofemoral OR femoral OR femoral artery*) adj3 (endarterectom* OR patch* OR repair*)) OR ((aortofemoral OR aortobifemoral OR femoral-distal OR femoral distal OR femoral-popliteal OR femoral popliteal OR femoral-tibial OR femoral tibial OR infrageniculate OR suprageniculate OR infrainguinal OR lower extremity OR lower limb OR peripheral vascular) adj3 (arterial surg* OR arterial bypass* OR bypass* OR bypass graft* OR bypass surg* OR graft* OR intervention* OR revascularization* OR revascularization procedure* OR vascular bypass* OR vascular bypass surg* OR vascular graft* OR vein graft* OR prosthetic graft*))	Artery bypass OR blood vessel graft OR bypass surgery OR critical limb ischemia/surgery OR endarterectomy OR limb ischemia/surgery OR peripheral artery occlusive disease/surgery OR prosthetic vascular graft OR vascular surgery	((iliofemoral OR femoral OR femoral artery*) adj3 (endarterectom* OR patch* OR repair*)) OR ((aortofemoral OR aortobifemoral OR femoral-distal OR femoral distal OR femoral-popliteal OR femoral popliteal OR femoral-tibial OR femoral tibial OR infrageniculate OR suprageniculate OR infrainguinal OR lower extremity OR lower limb OR peripheral vascular) adj3 (arterial surg* OR arterial bypass* OR bypass* OR bypass graft* OR bypass surg* OR graft* OR intervention* OR revascularization* OR revascularization procedure* OR vascular bypass* OR vascular bypass surg* OR vascular graft* OR vein graft* OR prosthetic graft*))
Infection	Infections OR surgical wound infection	infection* OR surgical site infection* OR surgical wound infection* OR wound infection*	Surgical infection OR wound infection	infection* OR surgical site infection* OR surgical wound infection* OR wound infection*

^a^MeSH: Medical Subject Heading.

### Data Management and Selection Process

The titles and abstracts of citations identified during the search will be imported into EndNote X9 reference management software (Clarivate, Thomson Reuters Corporation). This software will be used to remove identical duplicate citations before exporting them into Distiller SR (Evidence Reviews). Two investigators will then independently review the titles and abstracts of the articles identified by the search and select any article deemed potentially relevant by either investigator for full-text review. Finally, 2 investigators will review the full text of all potentially relevant citations and select studies for inclusion in the systematic review. Disagreements regarding study inclusion will be resolved via consensus or arbitration by a third investigator (Textbox 1).

Peripheral artery disease will be defined as intermittent vasculogenic claudication or chronic limb-threatening ischemia (ie, ischemic rest pain confirmed by vascular hemodynamic studies, lower limb ulceration, or gangrene) [6]. Lower limb revascularization surgery will be considered to include iliofemoral or femoral endarterectomy or patch angioplasty and aortofemoral, aortobifemoral, iliofemoral, femoral-popliteal, femoral-tibial, femoral-peroneal, axillofemoral, and femoral-femoral bypass. We will exclude studies that did not adjust for confounding in their effect estimates, exclusively examined certain high-risk patient cohorts (eg, those with obesity, diabetes, or chronic renal failure requiring dialysis), or included >20% of patients who underwent surgery for indications other than peripheral artery disease (eg, aneurysm disease). There will be no restrictions regarding the publication date, setting, or language of the study.

**Eligibility criteria.**

**Inclusion criteria**

- Population: The study included adults (aged ≥18 years) with peripheral artery disease who underwent lower limb revascularization surgery.
- Index and comparison prognostic factors: The study evaluated the prognostic value of a potential risk factor over and above (ie, adjusted for or independent of) other existing or comparator risk and confounding factors for predicting postoperative surgical site infection (SSI).
- Outcome, timing, and setting: The study reported odds ratios, risk ratios, or hazard ratios (and surrounding SE or 95% CIs) adjusted for the presence of other risk factors or confounding variables and relating the potential risk factors of interest to the development of SSI in patients undergoing lower limb revascularization surgery [18,19].
- Study design: Observational (ie, cohort or case-control) studies or secondary analyses of randomized controlled trial data.

### Outcomes

The primary outcomes will be early (in-hospital or ≤30 days) SSI and early Szilagyi grade I (cellulitis involving the wound), grade II (infection involving subcutaneous tissue), and grade III (infection involving the vascular graft) SSI. Secondary outcomes will be longer-term (>30 days) SSI or Szilagyi grade I (cellulitis involving the wound), grade II (infection involving subcutaneous tissue), or grade III (infection involving the vascular graft) SSI [17-19]. Although we will primarily use the Szilagyi classification to classify the severity of SSIs (as it is the most commonly used system in vascular surgery [18]), alternate classification systems for the severity of SSI are permitted if used by study authors (eg, the Centers for Disease Control, the American College of Surgeons National Surgical Quality Improvement Program, or the Veterans Affairs Quality Improvement Program).

### Data Items and Collection Process

Two investigators will independently extract data in duplicate using a predesigned electronic data extraction spreadsheet piloted on a representative sample of five included studies. We will extract the following data from the included studies: (1) design, data source, and setting of the study; (2) patient recruitment period; (3) patient and procedural characteristics, including the number and types of procedures performed, the proportion of patients who had a groin incision (vertical or oblique), and the indication for the procedure (ie, intermittent vasculogenic claudication or chronic limb-threatening ischemia); (4) reported potential risk factors for SSI; (5) reported adjusted associations between the reported risk factors and the development of SSI and Szilagyi grade I, grade II, and grade III SSI after lower limb revascularization surgery (or different severities of SSI defined using different classification systems); (6) other prognostic or confounding factors that were adjusted for when evaluating associations between potential risk factors and SSI (crude or unadjusted associations were not extracted); and (7) whether the authors adjusted for a minimum confounder set in their analyses. This minimum confounder set was defined based on a narrative review of published studies and will include surgical urgency, age, sex, obesity, diabetes, the presence of critical or chronic limb-threatening ischemia, and whether a groin incision was used.

### Risk of Bias Assessment

Two investigators will independently evaluate the study risk of bias in duplicate using the Quality in Prognosis Studies tool [38,39]. This tool includes questions regarding study participation and attrition, potential risk factor and outcome descriptions and measurements, confounding measurement and account, and methods and reporting of statistical analyses (the operationalized list of quality domains containing the prompting items used when making risk of bias decisions is given in Multimedia Appendix 2 [40]) [38,39]. The assessment of statistical analyses will incorporate recommendations for building and appraising logistic regression models [40,41]. We will assess whether studies that used administrative data used validated coding algorithms to identify indications for revascularization (eg, intermittent vasculogenic claudication or chronic limb-threatening ischemia), SSI, and SSI severity. Disagreements regarding risk of bias assessments will be resolved by consensus.

### Qualitative Data Synthesis

Before conducting quantitative data analyses, we will perform a narrative synthesis of candidate risk factors for SSI [42]. First, we will tabulate the reported risk factors along with the time of measurement of the SSI, the classification system used to determine the severity of SSI (where relevant), study data source, patient recruitment period, and study SSI outcome definition. This tabulation will be used to cluster reported risk factors into themes (eg, patient characteristics) and subthemes (eg, comorbidities) and identify potentially duplicate data. It will also allow us to identify risk factors for different severities of SSI after lower limb revascularization surgery that were classified using different severity classification systems but that have similar enough definitions to allow for meta-analysis. For example, we will combine risk factor estimates for Szilagyi grade I and grade II SSIs with those for superficial and deep incisional infections as defined by the Centers for Disease Control and American College of Surgeons National Quality Improvement Program.

### Quantitative Data Synthesis and Statistical Analyses

We will use the odds ratio as the summary measure of association for pooled analyses. Adjusted risk factor estimates with similar definitions will be pooled using DerSimonian and Laird random-effects models [43]. We will limit the primary analysis to prognostic studies that controlled for the minimum set of confounders, as recommended by guidance documents on meta-analyses of prognostic studies [34]. When adjusted risk factor estimates with the same definition were calculated from the same data source (eg, the American College of Surgeons National Surgical Quality Improvement Program) across several studies, we will include the estimate derived from the largest study. As a sensitivity analysis, we will also recalculate the estimate using that derived from the other smaller studies, as studies may have variably adjusted their estimates for potentially confounding factors.

We will inspect forest plots, calculate Cochran Q homogeneity and I^2^ inconsistency statistics, and conduct tests of homogeneity (*P<*.10 will be considered significant, given the low power of these tests) to assess for interstudy heterogeneity in the aforementioned estimates [44-46]. As suggested by Higgins et al [45], we will consider I^2^ statistics >25%, >50%, and >75% to represent low, moderate, and high degrees of heterogeneity, respectively. In the presence of at least low interstudy heterogeneity, we will conduct subgroup meta-analyses and meta-regression using DerSimonian and Laird random-effects models, with the summary odds ratio for SSI as the dependent variable. We will use the following predictor variables in an attempt to explain heterogeneity in these stratified meta-analyses and meta-regressions: (1) a high versus low risk of bias related to study participation and attrition, potential risk factor and outcome description and measurement, or methods and reporting of statistical analyses; (2) whether the potential risk factor was adjusted for the minimum confounder set; and (3) the type of lower limb revascularization surgery (stratified by aortofemoral or bifemoral bypass, axillofemoral or bifemoral bypass, a groin-only procedure, or an infrainguinal bypass). Finally, the proportion of patients undergoing an urgent surgical procedure or with these different types of lower limb revascularization surgeries will also be included as predictor variables in meta-regressions.

We will evaluate for evidence of small study effects potentially due to publication bias for each potential risk factor–SSI association by visually inspecting produced funnel plots and using Begg and Egger tests *(P<*.05 will be considered significant) [47]. Statistical analyses will be performed by a trained meta-analyst using Stata MP version 13.1 (Stata Corporation).

### Certainty in the Cumulative Evidence

We will use the Grading of Recommendations, Assessment, Development, and Evaluation (GRADE) approach for the assessment of evidence about prognostic factors to determine the certainty in the estimates of association between the reported risk factors and the development of SSI [48]. To do this, we will first assess the risk of bias, imprecision, inconsistency, indirectness, and publication bias associated with the evidence for the reported risk factors [49-53]. The overall certainty in these estimates will then be adjudicated as high (*further research is very unlikely to change our confidence in the estimate of effect*), moderate (*further research is likely to have an important impact on our confidence in the estimate of effect and may change the estimate*), low (*further research is very likely to have an important impact on our confidence in the estimate of effect and is likely to change the estimate*), or very low (*very uncertain about the estimate of effect*) [33].

## Results

As of June 10, 2021, we have submitted the search strategy to another medical librarian or information scientist to peer review it using the Peer Review of Electronic Search Strategies guideline [37]. We will execute the peer-reviewed search strategy on June 30 and then complete the review of titles and abstracts and full-text articles by July 30, 2021, and September 15, 2021, respectively. We will complete the full-text study data extraction and risk of bias assessment by November 15, 2021. Subsequently, we will conduct the qualitative data synthesis, followed by the quantitative data synthesis and the Grading of Recommendations, Assessment, Development, and Evaluation assessment of the results by November 1, 2021 before drafting the manuscript. We anticipate that we will be able to submit the manuscript for peer review by January 30, 2022.

## Discussion

### Principal Findings

SSIs are one of the most important complications in vascular surgery [11]. This study will identify, synthesize, and determine the certainty in the cumulative evidence associated with reported risk factors for SSI after lower limb revascularization surgery in patients with peripheral artery disease. It will also determine whether risk factors vary by the type of procedure performed (eg, a groin-only procedure or an infrainguinal bypass) or when the SSI was measured to occur (ie, in-hospital or more delayed). Finally, it will examine whether risk factors vary by how well study investigators accounted for different study risks of bias or other confounding or risk factors or whether the SSI involves the skin, subcutaneous tissue, or vascular graft.

### Implications

Results will be used to help surgeons, patients, and authors of clinical practice statements and guidelines in deciding on the safety of open vascular surgery in patients with medically refractory intermittent claudication and in selecting between open and endovascular revascularization when patients with chronic limb-threatening ischemia are candidates for both. It will also assist clinicians and policy makers in deciding which patients may benefit most from interventions designed to prevent SSIs (eg, incisional negative-pressure wound therapy [54]) and in designing future research in this area. This will include the creation of a prediction tool for identifying those at high risk for SSI after lower limb revascularization surgery and studies determining whether interventions that modify these risk factors before, during, or after surgery reduce the risk of SSI in this vulnerable population. The results may also assist in creating eligibility criteria for future randomized controlled trials designed to determine whether novel interventions or pathways are efficacious in preventing SSIs in this patient population.

## Appendix

Multimedia Appendix 1 Completed PRISMA-P (Preferred Reporting Items in Systematic Reviews and Meta-Analyses-Protocols) checklist.

Multimedia Appendix 2 Operationalized list of quality domains containing the prompting items used when making risk of bias decisions.

## Abbreviations

GRADE: Grading of Recommendations, Assessment, Development, and Evaluation
PICOTS: Population, Index Prognostic Factor, Comparison of Prognostic Factors, Outcome, Timing, and Setting
PRISMA: Preferred Reporting Items in Systematic Reviews and Meta-Analyses
PRISMA-P: Preferred Reporting Items in Systematic Reviews and Meta-Analysis Protocols
PROSPERO: International Prospective Register of Systematic Reviews
SSI: surgical site infection
