# Supplementary material for: Risk Factors for Surgical Site Infection After Lower Limb Revascularization Surgery in Adults With Peripheral Artery Disease: Protocol for a Systematic Review and Meta-analysis
Source: JMIR Res Protoc. 2021 Sep 16;10(9):e28759. doi: 10.2196/28759 (PMC8485188; doi:10.2196/28759)
Supplement: Multimedia Appendix 2 [file resprot_v10i9e28759_app2.docx]

**Multimedia Appendix 2.** Operationalized and expanded QUIPS guidelines for assessing risk of bias in the included studies.

| **Variables** | **Bias Domains** | | | | | |
| --- | --- | --- | --- | --- | --- | --- |
| **Study Quality Domains** | **Study Participation** | **Study Attrition** | **Prognostic Factor Measurement** | **Outcome Measurement** | **Confounding Measurement and Account** | **Statistical Analyses** |
| Prompting items | There was adequate participation in the study by eligible patients  The methods of identifying the sample and period and place of recruitment were adequately described  Inclusion and exclusion criteria were adequately explained  There was an adequate description of the baseline study sample demographics, comorbidities, and types of lower extremity arterial revascularization surgery | The response rate (i.e., proportion of study sample providing outcome data) was adequate  Reasons for loss to follow-up were provided  The characteristics of participants lost to follow-up were adequately described  There were likely to be no important differences between key characteristics and outcomes in participants who completed the study versus those who did not | A clear and clinically useful definition/description of the potential risk factor was provided  The method of potential risk factor measurement was adequately valid and reliable  Continuous variables were reported or appropriate (i.e., not data-dependent) cut-offs were used  An adequate proportion of the study sample had complete data for the potential risk factor  Appropriate methods of imputation were used for missing potential risk factor data | A clear definition of surgical site infection was provided  The definition of surgical site infection provided was similar to the definition by Szilagyi *et al*.[^19^](#_ENREF_19)  The method of surgical site infection measurement used was adequately valid and reliable  The method and setting of outcome measurement appeared similar for all study patients | Important potential confounding variables were clearly defined and accounted for in the study design or in the analysis | There was sufficient information to assess the adequacy of the analysis  The selected regression model was adequate for the design of the study  The regression model building strategy was based largely on theory or previous study^a^ |
| **Ratings** | **Rating Definitions** | | | | | |
| High risk of bias | The relationship between the potential risk factor and surgical site infection is very likely to be different for participants and eligible nonparticipants | The relationship between potential risk factor and surgical site infection is very likely to be different for completing and noncompleting participants | The measurement of the potential risk factor is very likely to be different for different levels of the outcome of interest | The measurement of surgical site infection had the potential for misclassification and was very likely to be different related to the baseline level of the indication | The observed “effect” of the potential risk factor on surgical site infection is very likely to be distorted by another factor related to the indication and outcome | The reported results are very likely to be spurious or biased related to analysis or reporting |
| Moderate risk of bias | The relationship between the potential risk factor and surgical site infection may be different for participants and eligible nonparticipants | The relationship between the potential risk factor and surgical site infection may be different for completing and noncompleting participants | The measurement of the potential risk factor may be different for different levels of the outcome of interest | The measurement of surgical site infection had the potential for misclassification and may be different related to the baseline level of the indication | The observed “effect” of the potential risk factor on surgical site infection may be distorted by another factor related to the indication and outcome | The reported results may be spurious or biased related to analysis or reporting |
| Low risk of bias | The relationship between the potential risk factor and surgical site infection is unlikely to be different for participants and eligible nonparticipants | The relationship between the potential risk factor and surgical site infection is unlikely to be different for completing and noncompleting participants | The measurement of the outcome of the potential risk factor is unlikely to be different for different levels of the outcome of interest | The measurement of surgical site infection had the potential for misclassification and is unlikely to be different related to the baseline level of the indication | The observed “effect” of the potential risk factor on surgical site infection is unlikely to be distorted by another factor related to the indication and outcome | The reported results are unlikely to be spurious or biased related to analysis or reporting |

Where QUIPS indicates Quality in Prognosis Studies.

^a^For logistic regression, the modeling strategy appeared appropriate (consider whether there were sufficient events per variable, continuous variables were reported to conform to a linear gradient, potentially important interactions were tested for, assessments were made for collinearity, and if the model was validated or goodness of fit measures were conducted) and was reported in detail (consider the selection of predictor variables, the fitting procedure, and the reporting of measures of predictive criterion validity such as whether the 95% confidence interval surrounding the point estimate of the odds ratio was provided).[^41^](#_ENREF_41)
